# Photothermal ablation of bone metastasis of breast cancer using PEGylated multi-walled carbon nanotubes

**DOI:** 10.1038/srep11709

**Published:** 2015-06-30

**Authors:** Zhen Lin, Yi Liu, Xueming Ma, Shaoyu Hu, Jiawei Zhang, Qian Wu, Wenbin Ye, Siyuan Zhu, Dehong Yang, Dongbin Qu, Jianming Jiang

**Affiliations:** 1 Department of Spinal Surgery, Nanfang Hospital, Southern Medical University, Guangzhou, Guangdong 510515, China; 2 Department of Oral Implantology and Prosthetic Dentistry, Academic Centre for Dentistry Amsterdam (ACTA), MOVE Research Institute, VU University Amsterdam and University of Amsterdam, Amsterdam, Netherlands

## Abstract

This study investigates therapeutic efficacy of photothermal therapy (PTT) in an orthotropic xenograft model of bone metastasis of breast cancer. The near-infrared (NIR) irradiation on Multi-Walled Carbon Nanotubes (MWNTs) resulted in a rapid heat generation which increased with the MWNTs concentration up to 100 μg/ml. MWNTs alone exhibited no toxicity, but inclusion of MWNTs dramatically decreased cell viability when combined with laser irradiation. Thermographic observation revealed that treatment with 10 μg MWNTs followed by NIR laser irradiation resulted in a rapid increase in temperature up to 73.4±11.98 °C in an intraosseous model of bone metastasis of breast cancer. In addition, MWNTs plus NIR laser irradiation caused a remarkably greater suppression of tumor growth compared with treatment with either MWNTs injection or NIR irradiation alone, significantly reducing the amount of tumor-induced bone destruction. All these demonstrate the efficacy of PTT with MWNTs for bone metastasis of breast cancer.

Breast cancer, the second leading cause of cancer-related deaths among women worldwide, almost invariably metastasizes to bones in patients with advanced disease^1^,^2^,^3^. Bone metastases induced by breast cancer are responsible for considerable skeletal morbidity, including pathological fracture, neurological compression and hypercalcaemia^4^,^5^. Conventional therapies for bone metastatic foci of breast cancer, like chemotherapy or surgery, all have their own special disadvantages. Chemotherapy, the most widely applied therapy for bone metastases, is highly limited by high toxicity and drug resistance^6^. The rich microenvironment of bone marrow facilitates survival of cancer cells and mediates drug resistance^7^. Surgery is only suitable for removal of the tumors with well-defined and primary lesions but not for treatment of small, poorly defined metastases. Thermal ablation therapies for solid tumors recently emerged as an attractive alternative approach, because it is minimally invasive, extensively indicative and leads to minimal side effects compared with traditional therapies. Currently, several thermal methods have been used for treatment of bone metastases, including focused ultrasound^8^,^9^,^10^,^11^, microwave^12^ and radiofrequency ablation^13^, but their effectiveness is limited by nonspecific heating of target tissue and likely risk of healthy tissue injury^14^,^15^,^16^. Efforts have never ceased to explore novel or alternative approaches which may yield more effective control or even eradication of bone metastatic foci by surmounting the side effects of current therapies.

An emerging photothermal therapy (PTT) is advantageous over traditional thermal therapies because its energy source can be adjusted and shaped to provide relatively uniform distribution of heat according to the tumor volume. Therefore, PTT provides better photothermal ablation specific to tumor foci, resulting in a more effective and minimally invasive therapy which leads to few complications^17^,^18^,^19^. It uses near-infrared (NIR) light to activate photothermal agents in a favorable NIR region (700–1,100 nm) to generate high heat which kills malignant cells^20^.

Recently, Multi-Walled Carbon Nanotubes (MWNTs), a class of nanotubes, have been extensively investigated as a sort of promising photothermal agent for PTT of cancer. They were reported to induce apoptosis of human pancreatic adenocarcinoma cells (PANC-1) by NIR laser-induced photothermal effects^21^. Pluronic F-127 coated MWNTs have been shown to be a highly effective NIR hyperthermia agent in PTT^22^. MWNTs are nested, cylindrical graphene structures with a strong optical absorbance in the NIR region of light. They own excellent electrical antenna properties compared with single-walled carbon nanotubes (SWNTs) and nanoshells^22^. According to classical antenna theory, optical coupling of light to nanotubes will generate maximum effect when nanotube lengths are longer than half the wavelength of the incident light. The nanotubes become an electrical dipole for incident irradiation in this situation^23^. However, inconsistent with classical antenna theory, currents within the nanotubes have a significantly long dephasing time. So, the currents travel with rare scatter (ballistically on/within the tube) and the nanotubes become a kind of “super-antenna”, as reported by studies^22^,^24^. MWNTs have excellent dipole antennae with broad absorption spectra compared with the specific resonance absorptions of Single-Walled Carbon Nanotubes (SWNTs) and nanoshells, suggesting they can be activated by a larger spectrum of NIR light^25^,^26^. In addition, because they possess more available electrons for absorption per particle and have a larger surface compared with SWNTs, they can absorb more NIR irradiation^26^. Therefore, using MWNTs as a photothermal agent in PTT can reduce the amount of NIR radiation and consequently the risk of skin damage.

Previous studies described the therapeutic potentials of PTT using MWNTs plus NIR irradiation for various cancers, but there has been no study into the effects of PTT via MWNTs plus NIR irradiation on bone metastasis of breast cancer. Therefore, we hypothesized that NIR irradiation combined with MWNTs might be a promising alternative therapy suitable for bone metastatic foci induced by breast cancer. The present study was designed to determine the effectiveness of PTT with MWNTs in an intraosseous model of bone metastasis of breast cancer.

## Results

### Synthesis and characterization of MWNTs

**Figure 1:**
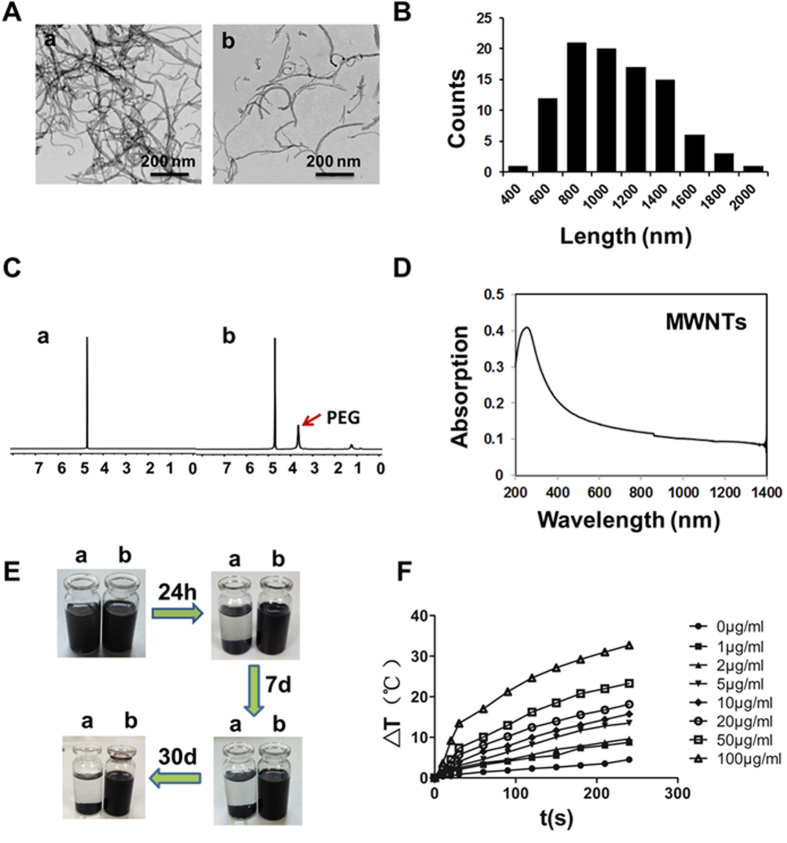
Characterization of MWNTs. (**A**) TEM images showed raw MWNTs (a) were aggregated in thick cords PBS at room temperature while PEGylated MWNTs (b) were mostly dissolved in the solution. Scale bars = 200 nm. (**B**) Histogram showing the size distribution of MWNTs obtained from TEM images. (**C**) The 1H NMR spectra of raw MWNTs (a) only showed the solvent residual peak while PEGylated MWNTs (b) showed PEG resonance peak and the solvent residual peak, indicating successful synthesis of PEGylated MWNTs. (**D**) Optical absorption measurement showed MWNTs had a high absorbance at a region of 700–1,100 nm. (**E**) The stability test of raw MWNTs (a) and PEGylated MWNTs (b) in PBS showed raw MWNTs gravitated to the bottom of the bottle after 24 h, but PEGylated MWNTs were stable even up to 30 days. (**F**) Evaluation of temperatures of MWNTs solutions clearly showed that the temperatures rose with the period of NIR irradiation (0–240 s) and also with the concentration of MWNTs (0–100  μg/ml) (n = 3).

As MWNTs have a highly hydrophobic surface, agglomeration will take place almost inevitably when they are dispersed in aqueous solutions. To stabilize the MWNTs in a solution, PEG coated MWNTs were prepared. The TEM images of MWNTs composites (Fig. 1A) demonstrated PEGylated MWNTs suspended individually in water in a single tube form while the raw MWNTs was winding up forming a large bundle. As shown in Fig. 1B, The average length of MWNTs was estimated to be 1126 ± 389 nm from 100 counts of MWNTs. In Fig. 1C showing the 1H NMR spectra (in D_2_O) of raw MWNTs and PEG functionalized MWNTs, PEG resonance peaks were clearly observed in the spectrum of PEG modified MWNTs, indicating successful synthesis of PEGylated MWNTs. MWNTs with a 1126 nm length resulted in a high absorbance at a region of 700–1,100 nm, as shown in Fig. 1D. In Fig. 1E showing the stability of raw MWNTs and PEGylated MWNTs, agglomeration and settlement occurred after raw MWNTs were dispersed in PBS for only 24 h, but PEGylated MWNTs were stably dissolved in PBS for 7 days or even up to weeks.

### MWNTs mediated photothermal effect generates significantly temperature elevation *in vitro*

To observe the photothermal effect of MWNTs *in vitro*, MWNTs solutions with different concentrations were irradiated with 808 nm laser at a power density of 5 W/cm^2^ with PBS as a control. MWNTs plus NIR laser produced a significantly greater amount of heat than laser alone (Fig. 1F). A temperature elevation of approximately 18 °C resulted from 1 min NIR irradiation on 100 μg/ml MWNTs while 1.5 °C from only 1 min NIR irradiation on PBS. Moreover, the temperature increase associated with MWNTs was positively related to exposure time. 4 min NIR irradiation elevated the temperature by 32.7 °C at a concentration of 100 μg/ml. In addition, the temperature of MWNTs also rose with the concentration of MWNTs (0–100 μg/ml) (Fig. 1F). These results indicated that MWNTs possessed a strong capacity of light-heat conversion under NIR laser irradiation.

### MWNTs have low cytotoxicity

**Figure 2:**
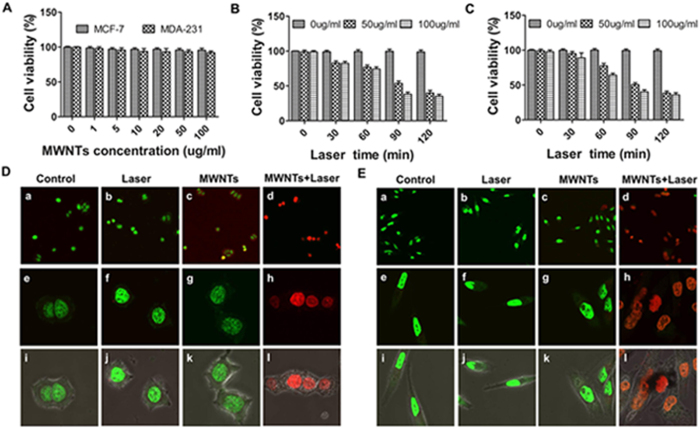
MWNTs mediated photothermal ablation efficiently kills cancer cells *in vitro*. (**A**) Viabilities of MCF-7 cells and MDA-231 showed relatively low toxicity when incubated with various concentrations of MWNTs (0–100 μg/ml). The cell viability was more than 95% even when the concentration of MWNTs was up to 100 μg/ml. The bars represent means ± s.d. (n = 3). Cell viabilities of MCF-7 (**B**) and MDA-231 (**C**) were measured by CCK8 after incubation with different concentrations of MWNTs and irradiated with NIR laser at 808 nm (5  W/cm^2^). MWNTs plus NIR irradiation significantly decreased the cell viability while neither the MWNTs nor the NIR irradiation alone destroyed cells. Moreover, the cell viability decreased with the duration of NIR irradiation. The bars represent means ± s.d. (n = 3). Live/dead cell assays for MCF-7 (**D**) and MDA-231 (**E**) cells treated with nothing (a, e, i), laser alone (b, f, j) MWNTs alone (c, g, k) and 100  μg/ml MWNTs + Laser (d, h, l). The photothermal effect of MWNTs plus laser (d, h, l) led to a large number of dead cells (red or yellow fluorescence), but cells treated with MWNTs alone (c, g, k), laser alone (b, f, j) and nothing (a, e, i) were mostly living (green fluorescence). Fluorescence microscopy images (i-l) showed overlapping green and red fluorescence on bright field images. Magnification ×200 (a-d); magnification ×600 (e-l).

The cytotoxicity of the MWNTs was evaluated in MCF-7 and MDA-231 cells using the CCK8 assay. The viability of untreated cells was about 100% and the cellular viability of MCF-7 cells remained above 95% when they were cultured with MWNTs of different concentrations (Fig. 2A). Similar situations were found in MDA-231 cells (Fig. 2A). The results indicated that MWNTs have low cytotoxicity.

### MWNTs mediated photothermal ablation efficiently kills cancer cells *in vitro*

CCK8 assay demonstrated that MWNTs combined with NIR irradiation (808 nm, 5 W/cm^2^) induced stronger cytotoxicity compared with either MWNTs or NIR light irradiation alone (Fig. 2B&C). 1 min 808 nm NIR irradiation at 5 W/cm^2^ did not change the viability of the tumor cells, but the viability of the tumor cells decreased significantly to 74.3% when treated with NIR irradiation combined with 100 μg/ml MWNTs. Moreover, the viability of the tumor cells decreased significantly as the NIR laser irradiation time increased. After 2 min NIR irradiation, the viability of the MCF-7 cells exposed to MWNTs at a concentration of 100 μg/ml dramatically decreased to 35.2% (Fig. 2B). The same trend was observed in 50 μg/ml MWNTs plus NIR irradiation (Fig. 2B). The situations in MDA-231 cells were similar (Fig. 2C). These demonstrated that MWNTs might be used as an effective photothermal agent for photothermal destruction of cancer cells.

To further assess the photothermal effect of MWNTs combined with NIR irradiation, cells were stained with Live-Dead cell staining kit which can distinguish live cells (green fluorescence) from dead or dying ones (red fluorescence). The majority of cells treated with either MWNTs alone or NIR irradiation alone (808 nm, 5 W/cm^2^) were living ones (green fluorescence), but a large number of cells treated with MWNTs plus NIR laser irradiation (808 nm, 5 W/cm^2^) were dead ones (red or yellowish fluorescence) (Fig. 2D,E). Similar morphological damage to MCF-7 (Fig. 2D i-l) and MDA-231 cells (Fig. 2E i-l) observed by confocal microscopy were shown by merging green and red fluorescence on bright field images. These results suggest that combination of MWNTs and NIR irradiation is necessary to achieve a lethal effect on tumor cells.

### MWNTs mediated photothermal effect generates significantly temperature elevation *in vivo*

**Figure 3:**
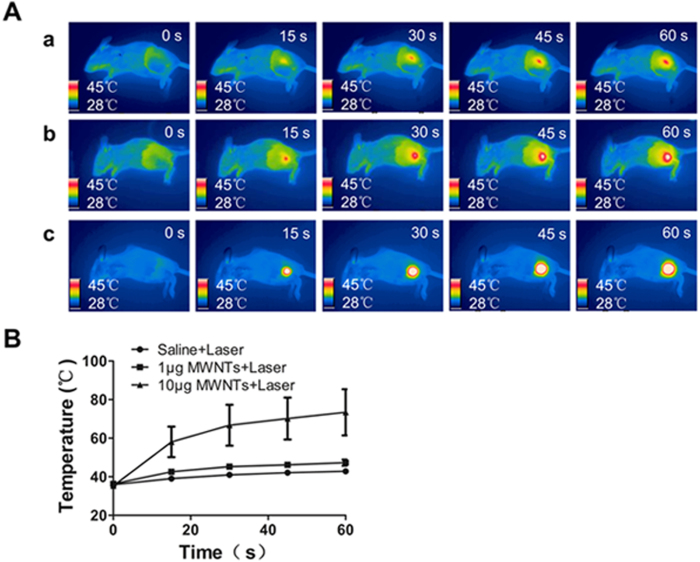
MWNTs mediated photothermal effect generates significantly temperature elevation *in vivo*. The infrared thermal images (**A**) and the linear graph (**B**) showed the temperature changes at the tumor site treated with saline + laser (row a), 1 μg MWNTs + NIR irradiation (row b), and 10 μg MWNTs + NIR irradiation (row c) at 0, 15, 30, 45 and 60 s respectively. MWNTs plus NIR irradiation caused dramatic temperature elevation compared with NIR irradiation alone. Additionally, prolonged NIR irradiation duration enhanced the temperature elevation. Moreover, temperature elevation was associated with the dosage of MWNTs. The bars represent means ± s.d. (n = 4).

We evaluated the photothermal effect of MWNTs *in vivo* by temperature monitoring using an infrared thermal imaging camera. As shown in Fig. 3A,B, laser irradiation (808 nm, 5 W/cm^2^) combined with MWNTs led to significantly higher temperatures than irradiation alone. After 60 s of laser irradiation, the temperature on the tumor surface increased rapidly up to 73.4 ± 11.98 °C in the 10 μg MWNTs plus laser group, to 47.3 ± 1.63 °C in the 1 μg MWNTs plus laser group, but only to 42.8 ± 1.10 °C in the laser only group. Additionally, the temperature increase at the tumor site was associated with the concentration of MWNTs (Fig. 3B). 10 μg MWNTs plus laser treatment caused a higher temperature increase at 60 s compared with the other two treatments (P < 0.001 vs laser group & P = 0.02 vs 1 μg MWNTs plus laser group). Moreover, the temperature increase at the tumor site was associated with the duration of NIR irradiation. In the 10 μg MWNTs plus laser group, the temperatures after irradiation for 15 s, 30 s, 45 s and 60 s at the tumor surface were constantly increased (Fig. 3B).

### MWNTs mediated photothermal ablation reduces tumor volume and cancer-induced bone destruction

**Figure 4:**
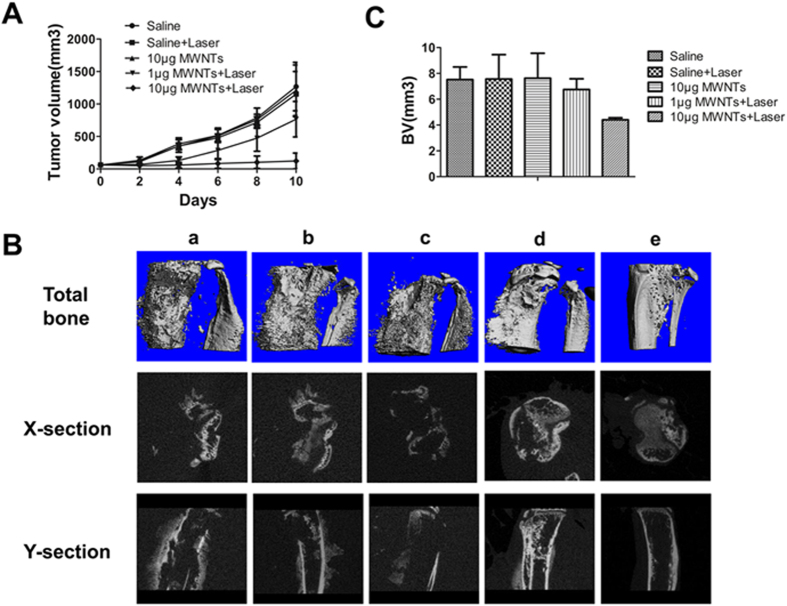
MWNTs mediated photothermal ablation reduces tumor volume and cancer-induced bone destruction. (**A**) Tumor growth curves for mice treated with saline, laser alone, 10 μg MWNTs, 1 μg MWNTs + laser, and 10 μg MWNTs + laser. There were no statistically significant differences (P > 0.05) in the mean tumor volume among the groups treated with saline, laser alone and 10 μg MWNTs, but tumor volume in the group treated with 10 μg MWNTs + laser was significantly smaller (P < 0.01). The effect of suppression tumor growth was attenuated with decreased dosage of MWNTs of 1 μg. 1 μg MWNTs + laser produced modest suppression in tumor volume, which was significantly larger than that in the group of 10 μg MWNTs + laser (P < 0.05). The bars represent means ± s.d. (n = 5). (**B**) Micro-CT images of tumor-bearing tibiae showed changes of bone structure subjected to treatment with saline (rank a), laser alone (rank b), (c) 10 μg MWNTs (rank c), 1 μg MWNTs + laser (rank d) and 10 μg MWNTs + laser (rank e) from total bone, transverse-section (X-section) and cross-section (Y-section) views respectively. In the groups treated with saline (a), saline + laser (b) and 10 μg MWNTs (c), the bone structure was all severely destroyed and massive osteosclerotic growth was observed. However, in the groups treated with 1 μg MWNTs + laser (d), and 10 μg MWNTs + laser (e), there was only mild bone destruction. Especially in the groups treated with 10 μg MWNTs + laser, the bone structure was significantly protected. (**C**) Tumor-bearing tibiae treated with 10 μg MWNTs + laser showed a statistically significant decrease in bone volume compared with those treated with saline, laser alone and 10 μg MWNTs (P < 0.05), but 1 μg MWNTs + laser led to only a slight reduction in bone volume compared with the other three groups (P > 0.05). The bars represent means ± s.d. (n = 5).

We next investigated the effectiveness of MWNTs-induced photothermal ablation in reducing tumor growth and cancer-induced bone destruction. As expected, MWNTs plus NIR irradiation (808 nm, 5 W/cm^2^) significantly inhibited tumor growth compared with MWNTs alone or NIR irradiation alone (Fig. 4A). There were no statistically significant differences regarding the mean tumor volume among the saline, NIR irradiation and MWNTs groups on day 10 after treatment (P > 0.05). However, the mean tumor volume in the 10 μg MWNTs plus NIR irradiation group on day 10 after treatment (122.4 ± 120.9 mm^3^) was significantly smaller than that in the saline group (1267.5 ± 327.5 mm^3^; P < 0.001). In addition, when the dosage of MWNTs dropped to 1 μg, their effect on suppressing tumor growth was greatly attenuated under the same NIR irradiation, indicating an association between the dosage and antitumor effect of MWNTs under NIR irradiation.

Qualitative assessment of bone architecture showed the bone structure was protected completely in the group treated with 10 μg MWNTs plus NIR irradiation (808 nm, 5 W/cm^2^). The group treated with 1 μg MWNTs plus NIR irradiation (808 nm, 5 W/cm^2^) showed partial destruction of cortical bone and new bone extending from the cortex. However, apparent osteolytic destruction and massive new bone formation was observed in the saline, NIR irradiation (808 nm, 5 W/cm^2^) and MWNTs groups (Fig. 4B). The bone volume of tumor-bearing tibia in the 10 μg MWNTs plus NIR irradiation group was significantly smaller than that in the saline, NIR irradiation and MWNTs groups (P < 0.05) (Fig. 4C). However, the 1 μg MWNTs plus NIR irradiation only slightly suppressed bone destruction, with no significant difference in bone volume from the saline, NIR irradiation or MWNTs group. (P > 0.05) (Fig. 4C).

### MWNTs mediated photothermal ablation does not affect tactile allodynia and body weight in mice

**Figure 5:**
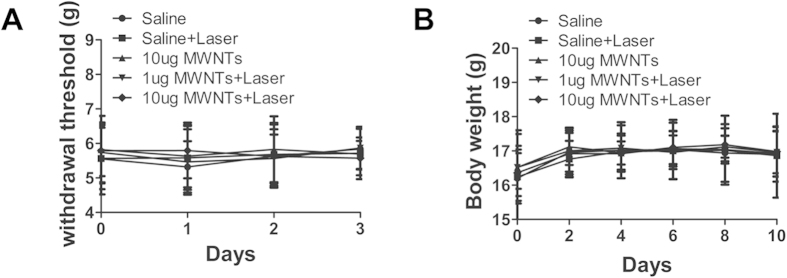
MWNTs mediated photothermal ablation does not affect tactile allodynia and body weight of mice. (**A**) The graph showed no apparent differences between the four measurements before treatment (0), one (1), two (2) and three (3) days after treatment respectively. The bars represent means ± s.d. (n = 5). (**B**) Six measurements showed the mice in the five groups gained similar body weight before treatment (0), two (2), four (4), six (6), eight (8) and ten (10) days after treatment respectively. The bars represent means ± s.d. (n = 5).

Previous study showed that thermal ablation resulted in neurodestruction and behavioral hypersensitivity to allodynia^27^. We therefore tested the effects of photothermal therapy of MWNTs on the tactile allodynia in mice using nociceptive tests. The maximum tactile allodynia in five animal groups was similar before treatment, and there were no significant between-group differences in the maximum tactile allodynia after photothermal therapy (Fig. 5A) (P > 0.05). We further monitored the body weights following treatments in mice. As expected, the animals had no significant body weight loss after treatment with MWNTs plus laser at the final follow-up (Fig. 5B). These results clearly indicated that MWNTs combined with NIR irradiation did not affect the withdrawal threshold or body weight, thus implying the PTT might be a safe therapy used in tumor-bearing mice.

## Discussion

Our current work demonstrated that PTT via MWNTs plus NIR irradiation effectively generated great heat which led to significant damage to MCF-7 and MDA-231 cells cultured *in vitro*. In addition, MWNTs plus NIR irradiation significantly increased the local temperature at the bone metastatic foci, reduced the tumor size in the mice, and protected the bone from cancer-induced destruction in an intraosseous model of bone metastasis of breast cancer. We further found that duration of NIR irradiation and dosage of MWNTs might be critical to maximum tumor destruction. Prolonged duration of NIR irradiation and increased dosage of MWNTs enhanced photothermal effect of PTT. Significantly, our therapy for the bone metastasis of breast cancer, MWNTs plus NIR irradiation, achieved high antitumor efficacy without significant side effects, as proved by nociceptive tests and body weight measurements.

NIR light at a region of 700–1,100 nm in wavelength is often used in PTT because it penetrates deeply into the tissue and is hardly absorbed by normal tissue^18^. In our study, we demonstrated that MWNTs show a high degree of absorption in this NIR region in the optical absorption measurement, which is consistent with previous studies^22^. Study show that MWNTs combined with 808 nm NIR light induce highly effective photothermal effect^21^. So, we chose 808 nm NIR light to stimulate MWNTs.

Neither MWNTs nor NIR irradiation alone resulted in cell destruction. MWNTs alone led to relatively low toxicity, with less than 5% cell damage under a concentration of 100 μg/ml. NIR irradiation alone at 5  W/cm^2^ for 2 min only caused minimal temperature elevation and no change in cell viability. However, MWNTs combined with NIR irradiation at 5  W/cm^2^ for 2 min dramatically enhanced the temperature and killed a large number of cells. Previous studies indicated that if NIR irradiation was used alone, the laser needed to be as high as 50.9 W/cm^2^ and prolonged to 10 min for destruction of the majority of cells^22^. Therefore, our study has demonstrated that MWNTs used together with NIR irradiation can significantly enhance the efficiency of heat generation, implying their role as a robust photothermal agent in tumor PTT.

Although inclusion of MWNTs is important for generation of high heat in PTT, irradiation duration is also an essential factor. In combination with 100 μg/ml MWNTs, NIR irradiation at 5  W/cm^2^ for 30 s increased the temperature to 66.7 °C, but 60 s NIR irradiation enhanced the temperature to 73.4 °C. Prolonged irradiation may allow MWNTs to absorb more NIR laser to generate more heat, leading to a maximum damage to cancer cells.

Moreover, antitumor capacity of PTT was associated with the dosage of MWNTs delivered to the tumor. In combination with NIR irradiation, 10 μg MWNTs yielded dramatical temperature elevation and significant suppress of tumor growth, showing a statistical significance compared with the saline group. However, 1 μg MWNTs only caused mild temperature increase and modest suppress of tumor growth, displaying no statistical significance compared with the saline group. This is consistent with a previous study, which presented a dose-dependent effect of MWNTs on tumor regression^26^.

Our photothermal ablation experiment revealed that 10 μg MWNTs plus NIR irradiation resulted in dramatically temperature increase of 73.4 ± 11.98 °C in 60 s. Although the temperature was far above the damage threshold of 43 °C supposed to cause irreversible damage to cancer cells^28^, the tumors were still not completely eradicated. We think this might be associated with two possible factors. First, the dispersibility of MWNTs in the tumor might not be good enough. The poor dispersibility of the photothermal agent was also observed by Hashida *et al.* who indicated that the poor dispersibility of nanotubes in tumor after intra-tumor injection might undermine the effect of PTT^29^. Secondly, since the bone metastasis induced by breast cancer had highly irregular margins it was hard to eliminate the remaining tumor cells thoroughly. As we know, if even a trace of tumor cells survive PTT treatment they can regrow to form a tumor tissue in a short period. Therefore, in order to completely eradicate cancer cells, it is necessary to optimize the parameters of PTT, such as NIR irradiation time, dosage of photothermal agent and dispersibility of photothermal agent. PTT combined with other therapeutic algorithms is another approach to enhancing the therapeutic effect.

Our study has other limitations. First, our animal models of bone metastasis induced by breast cancer were established by direct inoculation of tumor cells into the tibia of the mice. This, of course, did not simulate the steps of metastatic lesion spreading from the primary tumor to bone. However, our models allowed for relatively consistent tumor burden^30^ and a lesion location suitable for PTT. Secondly, we showed that the temperature rose with the period of NIR irradiation (0–4 min) and also with the concentration of MWNTs (0–100 μg/ml), but we did not determine the optimal irradiation duration and the optimal concentration of MWNTs which might lead to the maximal therapeutic efficacy. Optimization of all parameters for PTT using MWNTs plus NIR irradiation is a huge challenge for further studies. Thirdly, although we studied the effects of MWNTs-associated photothermal therapy on tumor growth and cancer-induced bone destruction, we did not address the effects on other tumor-related factors, like macrophages. According to Yang *et al.*, macrophages influenced by MWNTs plays an important role in the tumor progression and metastasis^31^. Thus, we should take macrophages into account in the further research.

In conclusion, our experiments demonstrates that MWNTs plus NIR laser irradiation is a promising therapeutic alternative because it is safe and effective for bone metastatic foci induced by breast cancer. Of course, many problems related to this treatment warrant extensive research before it can be clinically applied.

## Methods

### Materials

MWNTs (95% purity) with 20–30 nm in diameter and 0.5–2 μm in length were obtained from Chengdu Organic Chemicals Co. Ltd. (Sichuan, China). 1,2-distearoyl-snglycero-3-phosphoethanolamine-N-[methoxy(polyethyleneglycol)-2000] (DSPE-PEG2000) was purchased from Corden Pharma Switzerland LLC (Liestal, Switzerland). Cell Counting Kit-8 (CCK8) was purchased from Panera (Guangzhou, China). RPMI1640, DMEM, fetal bovine serum (FBS) were purchased from Gibco (Tulsa, OK, USA). All the other chemicals were of analytical grade, and purified water was produced by a millipore water purification system.

### Preparation of PEGylated MWNTs

PEGylated MWNTs were prepared by sonicating MWNTs with DSPE-PEG 2000 in aqueous media. 5 mg of MWNTs and 5 mg of DSPE-PEG2000 were put into a test tube and dispersed in 5 ml of water before sonication was performed in an ultrasonic bath for 2 h at room temperature. The PEGylated MWNTs were obtained in a millipore ultrafiltration tube with a molecular weight cutoff (MWCO) of 100 k Da and re-dispersed in PBS.

### Characterization of MWNTs

Samples of raw MWNTs and PEG functionalized MWNTs were scanned by a transmission electron microscope (Hitachi, Japan) at an acceleration voltage of 80 kV. The sizes of MWNTs on TEM images were measured using image analysis software, Image J (Ver. 1.48). The 1H NMR of raw MWNTs and PEG functionalized MWNTs were recorded at 400 Hz (AVANCE III, Bruker, Switzerland). Optical absorption of MWNTs (10 μg/ml) was measured with a Lambda 950 spectrophotometer (PerkinElmer, Inc.) in a wavelength range of 200 to 1,400 nm. The absorbance of deionized water was set as a zero baseline.

### Photothermal effect of MWNTs *in vitro*

To evaluate photothermal properties of MWNTs, temperature changes were measured. One milliliter of MWNTs solution at different concentrations (0–100 μg/ml) in a vial with a diameter of 1.6 cm and cross-section of 2.0 cm^2^ was irradiated with an NIR laser of 1.25 W (808 nm) (Changchun Femtosecond Technology Co., LTD, Liaoning, China) over an exposure area of 0.25 cm^2^ (5  W/cm^2^). The temperature increase was monitored every 30 s with a thermocouple immersed in the solution for 0–4 min.

### Cytotoxicity assay for MWNTs

Cell viability was assessed by a Cell Counting Kit-8 (CCK8) assay (Pantric Molecular Technologies, Guangzhou, China). MCF-7 and MDA-231 cells were seeded onto 96-well plates with a density of 10,000 cells/well and incubated for 24 h. Then 100 μl of MWNTs with different concentrations (0–100 μg/ml) were added to each well. After incubation for 24 h, 10 μl aliquot of CCK8 solution was added to each well and the cells were incubated for 2 h at 37 °C. The absorbance at 450 nm was measured with a microplate reader (SpectraMax M5; Molecular Devices, Sunnyvale, CA, USA). The cell viability percentage was determined relative to that of the control.

### MWNTs mediated Photothermal toxicity *in vitro*

The damage to tumor cells induced by thermal ablation with MWNTs and NIR irradiation was evaluated *in vitro*. MCF-7 and MDA-231 cells (1.0 × 10^4^ cells/100 μl) were seeded onto 96-well plates for 24 hours and then incubated with various concentrations of MWNTs for 24 hours. The cells were exposed to irradiation with an 808-nm NIR laser at 5 W/cm^2^ for 0-2 min. Cell survival efficiency was measured using the CCK8 assay as mentioned above at 16 hours post-irradiation.

To further verify the photothermal effect on cancer cells, the cells were stained with Live-Dead cell staining kit (Biovision, Mountain View, CA, US) at 16 hours after the photothermal treatment. Briefly, MCF-7 and MDA-231 cells (1.0 × 10^4^ cells/150 μl) were seeded onto confocal dishes (NEST Biotechnology Co. LTD, Jiangsu, China) and incubated overnight. Then 100 μg/ml MWNTs were added to the culture medium. After 24 h incubation, the cells were exposed to irradiation with an 808-nm NIR laser for 2 min at 5 W/cm^2^ and stained with Live-Dead cell staining kit 16 hours later. Briefly, cells were stained with 0.5 ml staining solution containing 0.5 μl Live-Dye (1 mM) and 0.5 μl PI (2.5 mg/ml). After incubated for 15 min at 37 °C, signals were visualized by confocal microscopy (FV10i-W, Olympus, Tokyo, Japan).

### Animals

*In vivo* experiments were conducted using female Balb/c, weighing approximately 18 g (5-week-old), obtained from Southern Medical University Experimental Animal Center (Guangzhou, China). Animals had free access to chow and water in a 25 °C ± 1 °C environment with regular light and 55% ± 5% relative humidity. Experiments were conducted in accordance with Guiding Principles for the Care and Use of Experiment Animals in Southern Medical University. The animal study protocols were approved by the Institutional Animal Care and Use Committee at Southern Medical University (NFYY-2014-65).

### Photothermal effect of MWNTs *in vivo*

The temperature changes in tumor sites were imaged by an infrared thermal imaging camera (FLIR T610, FLIR Systems Inc, Sweden). EMT6 cells were used to establish the tumor model because of their high rate of tumor formation in normal Balb/c mice. Single-cell suspensions of 1.0 × 10^6^ murine breast cancer EMT6 cells were injected into the tibiae of female Balb/c mice of 5-week-old. After 8 days of tumor inoculation, the mice were randomized into three different groups subjected to treatments by (i) saline (100 μl) + laser, (ii) MWNTs (100 μl, 10 μg/ml) + laser, (iii) MWNTs (100 μl, 100 μg/ml) + laser. One day later, NIR laser irradiation was carried out for 60 s with an 808 nm NIR laser at 1.25 W (5 W/cm^2^) under sodium pentobarbital anesthesia. During irradiation, the temperature changes in the tumor region were imaged using an infrared thermal imaging camera. Pictures were edited with FLIR QuickReports 1.2.

### *In vivo* therapeutic effect of MWNTs with NIR irradiation

The therapeutic effect of MWNTs and NIR laser irradiation was evaluated by measuring growth inhibition of the tumor inoculated in mice. Single-cell suspensions of 1.0 × 10^6^ murine breast cancer EMT6 cells were injected orthotopically into the tibiae of female Balb/c mice of 5-week-old. After 8 days of tumor inoculation, the mice were randomized into five different groups treated by (i) saline only (100 μl), (ii) saline (100 μl) + laser, (iii) MWNTs only (100 μl, 100 μg/ml), (iv) MWNTs (100 μl, 10 μg/ml) + laser, (v) MWNTs (100 μl, 100 μg/ml) + laser. At 24 h after the injection, the mice in the five groups were anesthetized for irradiation on the tumor sites with an NIR laser at 808 nm. The NIR laser treatment consisted of three rounds of 60 s of illumination at 1.25 W for 0.25 cm^2^ (5 W/cm^2^) with 30 s intervals. After NIR irradiation, the tumor sizes were measured by a caliper every other day and calculated as the volume = (tumor length) × (tumor width)^2^/2.

### Micro computed tomography

Excised formalin-fixed tibiae were scanned using a micro-CT system (μCT80, SCANCO MEDICAL, Switzerland) at a resolution of 18 μm, a tube voltage of 50 kV and a tube current of 0.1 mA. The volume of interest (VOI) was defined as 260 slices of approximately 4.7 mm in thickness starting from the growth plate of tibial interface and moving down the tibia. Bone volume was calculated to allow quantitative analysis of bone quality.

### Nociceptive tests

After PTT, the paw withdrawal latency was elicited with a hand-held force transducer (Electronic Von Frey Anesthesiometer, IITC Life Science, CA, US) adapted with a 0.5 mm^2^ polypropylene tip. Each animal was placed in an individual acrylic cage (12 × 10 × 17 cm) with a wire mesh floor and acclimatized to the cage for 15 minutes before the start of testing. An increasing force was applied perpendicular to the central area of the hindpaw with the polypropylene tip. The end-point was characterized by the removal of the paw followed by clear flinching movements. After paw withdrawal, the intensity of the pressure was automatically recorded.

### Statistical analysis

All data are presented as the mean ± standard deviation. Data presented were analyzed with one-way analysis of variance (ANOVA). P < 0.05 was accepted as a statistically significant difference compared to controls.

## Additional Information

**How to cite this article**: Lin, Z. *et al.* Photothermal ablation of bone metastasis of breast cancer using PEGylated multi-walled carbon nanotubes. *Sci. Rep.* **5**, 11709; doi: 10.1038/srep11709 (2015).

## References

[b1] Elsie K. M. *et al.* Current Knowledge, Attitudes and Practices of Women On Breast Cancer and Mammography at Mulago Hospital. Pan Afr Med J. 5, 9 (2010). 21120008 10.4314/pamj.v5i1.56186 PMC2984320

[b2] Caraglia M. *et al.* Emerging Anti-Cancer Molecular Mechanisms of Aminobisphosphonates. Endocr Relat Cancer. 13, 7–26 (2006). 16601276 10.1677/erc.1.01094

[b3] Janz N. K. *et al.* Population-Based Study of the Relationship of Treatment and Sociodemographics On Quality of Life for Early Stage Breast Cancer. QUAL LIFE RES. 14, 1467–1479 (2005). 16110927 10.1007/s11136-005-0288-6

[b4] Cicek M. & Oursler M. J. Breast Cancer Bone Metastasis and Current Small Therapeutics. Cancer Metastasis Rev. 25, 635–644 (2006). 17160709 10.1007/s10555-006-9035-x

[b5] Kozlow W. & Guise T. A. Breast Cancer Metastasis to Bone: Mechanisms of Osteolysis and Implications for Therapy. J Mammary Gland Biol Neoplasia. 10, 169–180 (2005). 16025223 10.1007/s10911-005-5399-8

[b6] Gonzalez-Angulo A. M., Morales-Vasquez F. & Hortobagyi G. N. Overview of Resistance to Systemic Therapy in Patients with Breast Cancer. ADV EXP MED BIOL. 608, 1–22 (2007). 17993229 10.1007/978-0-387-74039-3_1

[b7] Meads M. B., Hazlehurst L. A. & Dalton W. S. The Bone Marrow Microenvironment as a Tumor Sanctuary and Contributor to Drug Resistance. CLIN CANCER RES. 14, 2519–2526 (2008). 18451212 10.1158/1078-0432.CCR-07-2223

[b8] Stone J. & Matchett G. Combined Ultrasound and Fluoroscopic Guidance for Radiofrequency Ablation of the Obturator Nerve for Intractable Cancer-Associated Hip Pain. PAIN PHYSICIAN. 17, E83–E87 (2014). 24452660

[b9] Hurwitz M. D. *et al.* Magnetic Resonance-Guided Focused Ultrasound for Patients with Painful Bone Metastases: Phase III Trial Results. J Natl Cancer Inst. 106, 10.1093/jnci/dju082, (2014). PMC4112926 24760791

[b10] Napoli A. *et al.* MR Imaging-Guided Focused Ultrasound for Treatment of Bone Metastasis. RADIOGRAPHICS. 33, 1555–1568 (2013). 24108551 10.1148/rg.336125162

[b11] Liberman B. *et al.* Pain Palliation in Patients with Bone Metastases Using MR-guided Focused Ultrasound Surgery: A Multicenter Study. ANN SURG ONCOL. 16, 140–146 (2009). 19002530 10.1245/s10434-008-0011-2

[b12] Pusceddu C., Sotgia B., Fele R. M. & Melis L. Treatment of Bone Metastases with Microwave Thermal Ablation. J VASC INTERV RADIOL. 24, 229–233 (2013). 23200605 10.1016/j.jvir.2012.10.009

[b13] Lane M. D. *et al.* Combination Radiofrequency Ablation and Cementoplasty for Palliative Treatment of Painful Neoplastic Bone Metastasis: Experience with 53 Treated Lesions in 36 Patients. SKELETAL RADIOL. 40, 25–32 (2011). 20686765 10.1007/s00256-010-1010-5

[b14] Saldanha D. F. *et al.* Current Tumor Ablation Technologies: Basic Science and Device Review. Semin Intervent Radiol. 27, 247–254 (2010). 22550363 10.1055/s-0030-1261782 PMC3324188

[b15] Zhao Z. & Wu F. Minimally-Invasive Thermal Ablation of Early-Stage Breast Cancer: A Systemic Review. Eur J Surg Oncol. 36, 1149–1155 (2010). 20889281 10.1016/j.ejso.2010.09.012

[b16] Imamura J. *et al.* Neoplastic Seeding After Radiofrequency Ablation for Hepatocellular Carcinoma. AM J GASTROENTEROL. 103, 3057–3062 (2008). 19086957 10.1111/j.1572-0241.2008.02153.x

[b17] Green H. N. *et al.* A Histological Evaluation and *in Vivo* Assessment of Intratumoral Near Infrared Photothermal Nanotherapy-Induced Tumor Regression. Int J Nanomedicine. 9, 5093–5102 (2014). 25395847 10.2147/IJN.S60648 PMC4227627

[b18] Miao W., Shim G., Lee S. & Oh Y. K. Structure-Dependent Photothermal Anticancer Effects of Carbon-Based Photoresponsive Nanomaterials. BIOMATERIALS. 35, 4058–4065 (2014). 24508077 10.1016/j.biomaterials.2014.01.043

[b19] You J. *et al.* Effective Photothermal Chemotherapy Using Doxorubicin-Loaded Gold Nanospheres that Target EphB4 Receptors in Tumors. CANCER RES. 72, 4777–4786 (2012). 22865457 10.1158/0008-5472.CAN-12-1003 PMC3445780

[b20] Shen S. *et al.* CMCTS Stabilized Fe3O4 Particles with Extremely Low Toxicity as Highly Efficient Near-Infrared Photothermal Agents for *in Vivo* Tumor Ablation. NANOSCALE. 5, 8056–8066 (2013). 23873020 10.1039/c3nr01447a

[b21] Mocan T. *et al.* Photothermal Treatment of Human Pancreatic Cancer Using PEGylated Multi-Walled Carbon Nanotubes Induces Apoptosis by Triggering Mitochondrial Membrane Depolarization Mechanism. J CANCER. 5, 679–688 (2014). 25258649 10.7150/jca.9481 PMC4174512

[b22] Fisher J. W. *et al.* Photothermal Response of Human and Murine Cancer Cells to Multiwalled Carbon Nanotubes After Laser Irradiation. CANCER RES. 70, 9855–9864 (2010). 21098701 10.1158/0008-5472.CAN-10-0250 PMC3699181

[b23] Hanson G. W. Fundamental transmitting properties of carbon nanotube antennas. IEEE Trans Antennas Propag. 53, 3426–35 (2005).

[b24] Torti S. V. *et al.* Thermal Ablation Therapeutics Based On CN(x) Multi-Walled Nanotubes. Int J Nanomedicine. 2, 707–714 (2007). 18203437 PMC2676813

[b25] Hirsch L. R. *et al.* Nanoshell-Mediated Near-Infrared Thermal Therapy of Tumors Under Magnetic Resonance Guidance. Proc Natl Acad Sci U S A. 100, 13549–13554 (2003). 14597719 10.1073/pnas.2232479100 PMC263851

[b26] Burke A. *et al.* Long-Term Survival Following a Single Treatment of Kidney Tumors with Multiwalled Carbon Nanotubes and Near-Infrared Radiation. Proc Natl Acad Sci U S A. 106, 12897–12902 (2009). 19620717 10.1073/pnas.0905195106 PMC2722274

[b27] Perret D. M. *et al.* Application of pulsed radiofrequency currents to rat dorsal root ganglia modulates nerve injury-induced tactile allodynia. Anesth Analg. 113, 610–616 (2011). 21596869 10.1213/ANE.0b013e31821e974f PMC3196349

[b28] Hildebrandt B. *et al.* The Cellular and Molecular Basis of Hyperthermia. Crit Rev Oncol Hematol. 43, 33–56 (2002). 12098606 10.1016/s1040-8428(01)00179-2

[b29] Hashida Y. *et al.* Photothermal Ablation of Tumor Cells Using a Single-Walled Carbon Nanotube-Peptide Composite. J CONTROL RELEASE. 173, 59–66 (2014). 24211651 10.1016/j.jconrel.2013.10.039

[b30] Arrington S. A., Damron T. A., Mann K. A. & Allen M. J. Concurrent Administration of Zoledronic Acid and Irradiation Leads to Improved Bone Density, Biomechanical Strength, and Microarchitecture in a Mouse Model of Tumor-Induced Osteolysis. J SURG ONCOL. 97, 284–290 (2008). 18161868 10.1002/jso.20949

[b31] Yang M. *et al.* Multiwalled Carbon Nanotubes Interact with Macrophages and Influence Tumor Progression and Metastasis. Theranostics. 2, 258–270 (2012). 22509194 10.7150/thno.3629 PMC3326737

